# Propyl 2-(3-benzoyl­thio­ureido)acetate

**DOI:** 10.1107/S1600536808030596

**Published:** 2008-10-09

**Authors:** Ibrahim N. Hassan, Bohari M. Yamin, Mohammad B. Kassim

**Affiliations:** a School of Chemical Sciences and Food Technology, Faculty of Science and Technology, Universiti Kebangsaan Malaysia, UKM 43600 Bangi Selangor, Malaysia

## Abstract

The title compound, C_13_H_16_N_2_O_3_S, is a thio­urea derivative with benzoyl and propoxycarbonyl­methyl groups attached to the two terminal N atoms. These groups adopt *trans* and *cis* configurations, respectively, with respect to the S atom across the thio­urea C—N bonds. The compound crystallizes in the *P*2_1_/*c* space group with *Z* = 8, resulting in two unique molecules in the asymmetric unit linked by C—H⋯S and C—H⋯O hydrogen bonds, forming a one-dimensional zigzag chain along the *c* axis.

## Related literature

For information on bond lengths and angles, see: Allen *et al.* (1987 ▶). For related literature on an analogous molecule, see: Hassan *et al.* (2008 ▶). For related structures, see: Yamin & Hassan (2004 ▶); Yamin & Yusof (2003 ▶).
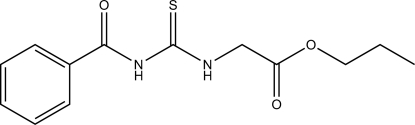


## Experimental

### 

#### Crystal data

C_13_H_16_N_2_O_3_S
*M* *_r_* = 280.34
Monoclinic, 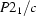

*a* = 11.6722 (19) Å
*b* = 15.105 (3) Å
*c* = 16.584 (3) Å
β = 104.737 (3)°
*V* = 2827.6 (8) Å^3^
*Z* = 8
Mo *K*α radiation
μ = 0.23 mm^−1^
*T* = 298 (2) K
0.34 × 0.29 × 0.09 mm

#### Data collection

Bruker SMART APEX CCD area-detector diffractometer
Absorption correction: multi-scan (*SADABS*; Bruker, 2000 ▶) *T*_min_ = 0.925, *T*_max_ = 0.979
15002 measured reflections
5262 independent reflections
2854 reflections with *I* > 2σ(*I*)
*R*_int_ = 0.045

#### Refinement

*R*[*F* ^2^ > 2σ(*F* ^2^)] = 0.068
*wR*(*F* ^2^) = 0.162
*S* = 1.05
5262 reflections
359 parameters
4 restraints
H atoms treated by a mixture of independent and constrained refinement
Δρ_max_ = 0.21 e Å^−3^
Δρ_min_ = −0.17 e Å^−3^

### 

Data collection: *SMART* (Bruker, 2000 ▶); cell refinement: *SAINT* (Bruker, 2000 ▶); data reduction: *SAINT*; program(s) used to solve structure: *SHELXS97* (Sheldrick, 2008 ▶); program(s) used to refine structure: *SHELXL97* (Sheldrick, 2008 ▶); molecular graphics: *SHELXTL* (Sheldrick, 2008 ▶); software used to prepare material for publication: *SHELXTL*, *PARST* (Nardelli, 1995 ▶) and *PLATON* (Spek, 2003 ▶).

## Supplementary Material

Crystal structure: contains datablocks global, I. DOI: 10.1107/S1600536808030596/at2635sup1.cif

Structure factors: contains datablocks I. DOI: 10.1107/S1600536808030596/at2635Isup2.hkl

Additional supplementary materials:  crystallographic information; 3D view; checkCIF report

## Figures and Tables

**Table 1:** Hydrogen-bond geometry (Å, °)

*D*—H⋯*A*	*D*—H	H⋯*A*	*D*⋯*A*	*D*—H⋯*A*
N2—H2*B*⋯O1	0.87 (2)	1.92 (2)	2.617 (3)	136 (2)
N2—H2*B*⋯O2	0.87 (2)	2.33 (2)	2.663 (4)	103.1 (17)
N4—H4*B*⋯O4	0.87 (3)	1.97 (2)	2.605 (4)	129 (3)
N4—H4*B*⋯O5	0.87 (3)	2.23 (3)	2.671 (4)	111 (2)
C5—H5*A*⋯S2	0.93	2.84	3.396 (3)	120
C13—H13*B*⋯O4^i^	0.96	2.54	3.329 (6)	139
C14—H14*A*⋯S1	0.93	2.78	3.397 (3)	125
C24—H24*A*⋯O2^ii^	0.97	2.57	3.441 (4)	150
C26—H26*B*⋯O1^ii^	0.96	2.57	3.384 (4)	143

Symmetry codes: (i) 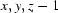; (ii) 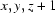.

**Fig. 1.**
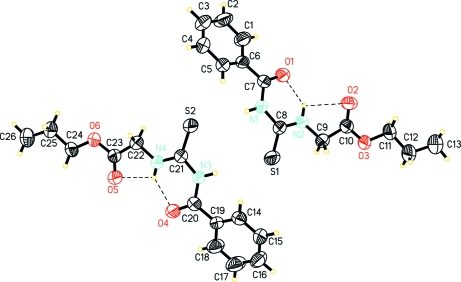
The molecular structure of (I), with displacement ellipsods are drawn at the 50% probability level.

**Fig. 2.**
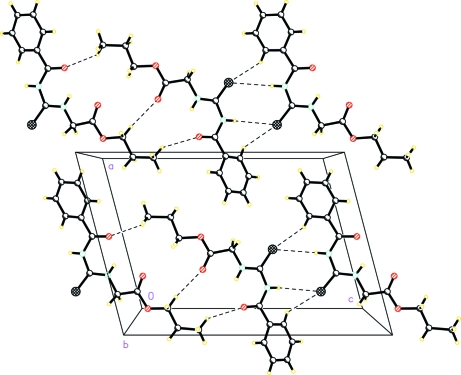
Crystal packing of (I) viewed down the a axis. Hydrogen bonds are drawn as dashed lines.

## supplementary crystallographic information

### Comment

The title compound, (I), is an analog of the previously reported ethyl 2-(3-benzoylthioureido) acetate (II) (Hassan *et al.*, 2008). As in most carbonylthiourea derivatives of the type *R*^1^C(O)NHC(S)NH*R*^2^, such as in N-benzoyl-N'-phenylthiourea (Yamin & Yusof, 2003) and 1-(2-morpholinoethyl)-3-(3-phenylacryloyl)thiourea (Yamin & Hassan, 2004), the molecule maintains its *cis*–*trans* configuration with respect to the positions of the propyl acetate and benzoyl groups, respectively, relative to the S atom across the C—N bonds (Fig. 1). The bond lengths and angles in the molecules are in normal ranges (Allen *et al.*, 1987) and comparable to those in (II). However, the C═S bond length [1.658 (3) Å] is slightly shorter than that of (II) [1.666 (2) Å]. The phenyl ring [C1–C6 (A)] and the carbonyl thiourea [(S1/N1/N2/O1/C7/C8/C9) (B)] fragments are essentially planar. In the acetate fragment, [O2/O3/C9/C10 (C)], the maximum deviation from the mean plane is 0.013 (3) Å for atom C10. The dihedral angles A/B and B/C are 18.58 (15)° and 20.51 (16)°, respectively. The phenyl ring is inclined to the acetate mean plane with a dihedral angle of 37.07 (19)°. The intramolecular hydrogen bonds N2—H2B···O1 and N2—H2B···O2 (Table 2) form a pseudo-six-member ring (N2/H2B/O1/C7/N1/C8) and pseudo-five-member ring (N2/H2B/O2/C10/C9), respectively. In the crystal structure, molecules are linked by intermolecular C—H···S, C—H···O and N—H···S hydrogen bonds (Table 2), forming a one-dimensional chain parallel to the *c* axis as seen in (II) (Fig. 2).

### Experimental

The title compound (I) was synthesized according to a previously reported compound (Hassan *et al.*, 2008). A yellowish crystal, suitable for X-ray crystallography, was obtained by a recrystallization from dichloromethane (yield 75%).

### Refinement

The C-bond H atoms were positioned geometrically and allowed to ride on their parent atoms, with *U*_iso_ = 1.2*U*_eq_ (C) for aromatic 0.93 Å, *U*_iso_ = 1.2*U*_eq_ (C) for CH_2_ 0.97 Å and *U*_iso_ = 1.5*U*_eq_ (C) for CH_3_ 0.96 Å. The H atoms of the amine groups were located in differece Fourier map and refined isotroplically with a restrained N—H distance of 0.87 (1) Å.

### Crystal data

**Table:**

C_13_H_16_N_2_O_3_S	*F*(000) = 1184
*M**_r_* = 280.34	*D*_x_ = 1.317 Mg m^−^^3^
Monoclinic, *P*2_1_/*c*	Mo *K*α radiation, λ = 0.71073 Å
Hall symbol: -P 2ybc	Cell parameters from 1848 reflections
*a* = 11.6722 (19) Å	θ = 1.8–25.5°
*b* = 15.105 (3) Å	µ = 0.23 mm^−^^1^
*c* = 16.584 (3) Å	*T* = 298 K
β = 104.737 (3)°	Block, yellowish
*V* = 2827.6 (8) Å^3^	0.34 × 0.29 × 0.09 mm
*Z* = 8	

### Data collection

**Table:**

Bruker SMART APEX CCD area-detector diffractometer	5262 independent reflections
Radiation source: fine-focus sealed tube	2854 reflections with *I* > 2σ(*I*)
graphite	*R*_int_ = 0.045
ω scans	θ_max_ = 25.5°, θ_min_ = 1.8°
Absorption correction: multi-scan (SADABS; Bruker, 2000)	*h* = −14→14
*T*_min_ = 0.925, *T*_max_ = 0.979	*k* = −14→18
15002 measured reflections	*l* = −19→20

### Refinement

**Table:**

Refinement on *F*^2^	Primary atom site location: structure-invariant direct methods
Least-squares matrix: full	Secondary atom site location: difference Fourier map
*R*[*F*^2^ > 2σ(*F*^2^)] = 0.068	Hydrogen site location: inferred from neighbouring sites
*wR*(*F*^2^) = 0.162	H atoms treated by a mixture of independent and constrained refinement
*S* = 1.05	*w* = 1/[σ^2^(*F*_o_^2^) + (0.0665*P*)^2^ + 0.3497*P*] where *P* = (*F*_o_^2^ + 2*F*_c_^2^)/3
5262 reflections	(Δ/σ)_max_ < 0.001
359 parameters	Δρ_max_ = 0.21 e Å^−^^3^
4 restraints	Δρ_min_ = −0.17 e Å^−^^3^

### Special details

**Table:**

Geometry. All e.s.d.'s (except the e.s.d. in the dihedral angle between two l.s. planes) are estimated using the full covariance matrix. The cell e.s.d.'s are taken into account individually in the estimation of e.s.d.'s in distances, angles and torsion angles; correlations between e.s.d.'s in cell parameters are only used when they are defined by crystal symmetry. An approximate (isotropic) treatment of cell e.s.d.'s is used for estimating e.s.d.'s involving l.s. planes.
Refinement. Refinement of *F*^2^ against ALL reflections. The weighted *R*-factor *wR* and goodness of fit *S* are based on *F*^2^, conventional *R*-factors *R* are based on *F*, with *F* set to zero for negative *F*^2^. The threshold expression of *F*^2^ > σ(*F*^2^) is used only for calculating *R*-factors(gt) *etc*. and is not relevant to the choice of reflections for refinement. *R*-factors based on *F*^2^ are statistically about twice as large as those based on *F*, and *R*- factors based on ALL data will be even larger.

### Fractional atomic coordinates and isotropic or equivalent isotropic displacement parameters (Å^2^)

**Table:**

	*x*	*y*	*z*	*U*_iso_*/*U*_eq_	
C1	−0.1076 (3)	0.8544 (3)	0.0140 (2)	0.0722 (11)	
H1A	−0.1024	0.8130	−0.0264	0.087*	
C2	−0.2152 (3)	0.8706 (3)	0.0310 (3)	0.0848 (13)	
H2A	−0.2822	0.8397	0.0024	0.102*	
C3	−0.2240 (3)	0.9320 (3)	0.0899 (3)	0.0768 (12)	
H3A	−0.2962	0.9418	0.1024	0.092*	
C4	−0.1257 (3)	0.9787 (3)	0.1302 (2)	0.0693 (11)	
H4A	−0.1319	1.0215	0.1692	0.083*	
C5	−0.0180 (3)	0.9630 (2)	0.1138 (2)	0.0574 (9)	
H5A	0.0481	0.9954	0.1413	0.069*	
C6	−0.0074 (3)	0.8993 (2)	0.05653 (19)	0.0496 (8)	
C7	0.1050 (3)	0.8780 (2)	0.0338 (2)	0.0497 (8)	
C8	0.3228 (3)	0.9015 (2)	0.0755 (2)	0.0516 (8)	
C9	0.4434 (3)	0.8520 (2)	−0.01556 (19)	0.0613 (10)	
H9A	0.4869	0.8037	0.0170	0.074*	
H9B	0.4912	0.9052	−0.0031	0.074*	
C10	0.4199 (3)	0.8311 (2)	−0.1062 (2)	0.0587 (9)	
C11	0.5118 (3)	0.7963 (3)	−0.2139 (2)	0.0736 (11)	
H11A	0.4604	0.8405	−0.2472	0.088*	
H11B	0.4796	0.7382	−0.2314	0.088*	
C12	0.6336 (4)	0.8042 (3)	−0.2255 (2)	0.0941 (14)	
H12A	0.6655	0.8618	−0.2060	0.113*	
H12B	0.6838	0.7596	−0.1919	0.113*	
C13	0.6363 (4)	0.7930 (4)	−0.3139 (3)	0.1222 (19)	
H13A	0.7164	0.7981	−0.3184	0.183*	
H13B	0.5885	0.8381	−0.3472	0.183*	
H13C	0.6056	0.7358	−0.3333	0.183*	
C14	0.6343 (3)	0.9685 (2)	0.3361 (2)	0.0616 (9)	
H14A	0.5673	0.9970	0.3044	0.074*	
C15	0.7394 (3)	0.9753 (3)	0.3139 (2)	0.0733 (11)	
H15A	0.7429	1.0078	0.2670	0.088*	
C16	0.8391 (3)	0.9346 (3)	0.3604 (3)	0.0784 (12)	
H16A	0.9099	0.9380	0.3446	0.094*	
C17	0.8334 (3)	0.8889 (3)	0.4306 (3)	0.0912 (13)	
H17A	0.9013	0.8626	0.4636	0.109*	
C18	0.7284 (3)	0.8816 (3)	0.4524 (2)	0.0773 (12)	
H18A	0.7256	0.8504	0.5002	0.093*	
C19	0.6270 (3)	0.9199 (2)	0.40472 (19)	0.0505 (8)	
C20	0.5155 (3)	0.9068 (2)	0.4304 (2)	0.0577 (9)	
C21	0.2955 (3)	0.9159 (2)	0.3802 (2)	0.0533 (9)	
C22	0.1736 (3)	0.8807 (2)	0.4749 (2)	0.0619 (10)	
H22A	0.1218	0.8419	0.4352	0.074*	
H22B	0.1356	0.9381	0.4727	0.074*	
C23	0.1951 (3)	0.8429 (2)	0.5608 (2)	0.0577 (9)	
C24	0.1014 (3)	0.8073 (3)	0.66818 (19)	0.0659 (10)	
H24A	0.1632	0.8375	0.7091	0.079*	
H24B	0.1196	0.7445	0.6705	0.079*	
C25	−0.0153 (3)	0.8223 (3)	0.6859 (2)	0.0830 (12)	
H25A	−0.0768	0.7951	0.6424	0.100*	
H25B	−0.0310	0.8854	0.6855	0.100*	
C26	−0.0200 (4)	0.7843 (3)	0.7694 (2)	0.1020 (15)	
H26A	−0.0966	0.7953	0.7787	0.153*	
H26B	0.0397	0.8119	0.8128	0.153*	
H26C	−0.0060	0.7217	0.7697	0.153*	
S1	0.43593 (8)	0.94060 (8)	0.14867 (6)	0.0800 (4)	
S2	0.18284 (8)	0.94066 (8)	0.30082 (6)	0.0798 (4)	
O1	0.10454 (18)	0.83491 (16)	−0.02851 (14)	0.0655 (7)	
O2	0.3254 (2)	0.8365 (2)	−0.15350 (16)	0.1033 (11)	
O3	0.51810 (19)	0.80959 (16)	−0.12641 (13)	0.0662 (7)	
O4	0.5165 (2)	0.88349 (19)	0.50080 (15)	0.0841 (9)	
O5	0.2888 (2)	0.81729 (17)	0.60138 (14)	0.0708 (7)	
O6	0.09492 (18)	0.84165 (16)	0.58525 (13)	0.0653 (7)	
N1	0.2084 (2)	0.90800 (19)	0.08632 (17)	0.0533 (7)	
N2	0.3327 (2)	0.8649 (2)	0.00618 (17)	0.0571 (8)	
N3	0.4114 (2)	0.92075 (19)	0.37143 (16)	0.0533 (7)	
N4	0.2860 (2)	0.8897 (2)	0.45399 (18)	0.0600 (8)	
H3B	0.416 (3)	0.935 (2)	0.3220 (10)	0.074 (12)*	
H2B	0.2684 (17)	0.847 (2)	−0.0292 (16)	0.070 (12)*	
H1B	0.206 (3)	0.9362 (19)	0.1312 (13)	0.069 (11)*	
H4B	0.346 (2)	0.870 (3)	0.4919 (18)	0.105 (16)*	

### Atomic displacement parameters (Å^2^)

**Table:**

	*U*^11^	*U*^22^	*U*^33^	*U*^12^	*U*^13^	*U*^23^
C1	0.051 (2)	0.085 (3)	0.079 (3)	0.003 (2)	0.014 (2)	−0.016 (2)
C2	0.041 (2)	0.104 (4)	0.107 (3)	−0.006 (2)	0.015 (2)	−0.014 (3)
C3	0.049 (2)	0.100 (3)	0.085 (3)	0.008 (2)	0.026 (2)	0.005 (3)
C4	0.056 (2)	0.096 (3)	0.061 (2)	0.004 (2)	0.0243 (19)	−0.002 (2)
C5	0.049 (2)	0.073 (3)	0.051 (2)	0.0037 (18)	0.0134 (16)	0.0041 (18)
C6	0.0412 (19)	0.059 (2)	0.048 (2)	0.0043 (17)	0.0091 (16)	0.0127 (17)
C7	0.046 (2)	0.058 (2)	0.044 (2)	0.0019 (17)	0.0083 (17)	0.0041 (17)
C8	0.0446 (19)	0.061 (2)	0.051 (2)	0.0042 (16)	0.0159 (16)	0.0015 (17)
C9	0.047 (2)	0.085 (3)	0.053 (2)	0.0110 (19)	0.0149 (17)	−0.0033 (18)
C10	0.053 (2)	0.073 (3)	0.050 (2)	0.010 (2)	0.0144 (19)	0.0010 (18)
C11	0.075 (3)	0.101 (3)	0.048 (2)	0.008 (2)	0.0221 (19)	0.001 (2)
C12	0.081 (3)	0.145 (4)	0.064 (3)	0.015 (3)	0.033 (2)	0.000 (2)
C13	0.102 (4)	0.199 (6)	0.075 (3)	−0.003 (4)	0.038 (3)	0.000 (3)
C14	0.051 (2)	0.081 (3)	0.049 (2)	−0.0051 (19)	0.0066 (17)	0.0075 (19)
C15	0.062 (2)	0.103 (3)	0.059 (2)	−0.019 (2)	0.023 (2)	0.001 (2)
C16	0.054 (2)	0.097 (3)	0.091 (3)	−0.007 (2)	0.031 (2)	−0.009 (3)
C17	0.051 (2)	0.111 (4)	0.107 (4)	0.010 (2)	0.010 (2)	0.030 (3)
C18	0.048 (2)	0.100 (3)	0.080 (3)	0.005 (2)	0.009 (2)	0.029 (2)
C19	0.0401 (18)	0.062 (2)	0.048 (2)	−0.0049 (17)	0.0094 (16)	−0.0031 (17)
C20	0.053 (2)	0.072 (3)	0.048 (2)	−0.0006 (18)	0.0109 (18)	0.0033 (18)
C21	0.0461 (19)	0.066 (2)	0.048 (2)	0.0012 (17)	0.0133 (16)	−0.0017 (17)
C22	0.0465 (19)	0.087 (3)	0.054 (2)	−0.0023 (19)	0.0160 (17)	0.0005 (19)
C23	0.050 (2)	0.073 (3)	0.052 (2)	−0.006 (2)	0.0149 (19)	−0.0118 (18)
C24	0.064 (2)	0.091 (3)	0.045 (2)	−0.007 (2)	0.0166 (18)	0.0012 (19)
C25	0.068 (3)	0.125 (4)	0.063 (3)	0.006 (2)	0.028 (2)	0.004 (2)
C26	0.099 (3)	0.152 (4)	0.065 (3)	−0.006 (3)	0.040 (2)	0.009 (3)
S1	0.0493 (5)	0.1229 (10)	0.0681 (7)	−0.0131 (6)	0.0158 (5)	−0.0340 (6)
S2	0.0485 (5)	0.1340 (10)	0.0562 (6)	0.0120 (6)	0.0123 (5)	0.0196 (6)
O1	0.0526 (14)	0.0873 (19)	0.0552 (15)	0.0002 (13)	0.0114 (12)	−0.0157 (13)
O2	0.0507 (16)	0.198 (3)	0.0565 (17)	0.0217 (19)	0.0055 (14)	−0.0217 (18)
O3	0.0530 (14)	0.100 (2)	0.0487 (15)	0.0152 (13)	0.0182 (11)	−0.0011 (12)
O4	0.0499 (15)	0.149 (3)	0.0520 (16)	−0.0021 (15)	0.0104 (12)	0.0278 (16)
O5	0.0522 (15)	0.102 (2)	0.0575 (16)	0.0037 (14)	0.0133 (12)	0.0067 (13)
O6	0.0513 (14)	0.100 (2)	0.0476 (14)	−0.0055 (13)	0.0177 (11)	0.0008 (12)
N1	0.0449 (16)	0.071 (2)	0.0453 (18)	0.0000 (15)	0.0131 (14)	−0.0076 (15)
N2	0.0401 (17)	0.085 (2)	0.0465 (18)	0.0048 (16)	0.0106 (15)	−0.0061 (15)
N3	0.0414 (16)	0.077 (2)	0.0415 (17)	0.0009 (14)	0.0106 (14)	0.0073 (15)
N4	0.0470 (17)	0.090 (2)	0.0461 (18)	−0.0001 (17)	0.0174 (15)	0.0069 (16)

### Geometric parameters (Å, °)

**Table:**

C1—C2	1.378 (5)	C14—H14A	0.9300
C1—C6	1.380 (4)	C15—C16	1.367 (5)
C1—H1A	0.9300	C15—H15A	0.9300
C2—C3	1.368 (5)	C16—C17	1.370 (5)
C2—H2A	0.9300	C16—H16A	0.9300
C3—C4	1.368 (5)	C17—C18	1.368 (5)
C3—H3A	0.9300	C17—H17A	0.9300
C4—C5	1.373 (4)	C18—C19	1.373 (4)
C4—H4A	0.9300	C18—H18A	0.9300
C5—C6	1.378 (4)	C19—C20	1.482 (4)
C5—H5A	0.9300	C20—O4	1.217 (4)
C6—C7	1.490 (4)	C20—N3	1.369 (4)
C7—O1	1.220 (3)	C21—N4	1.317 (4)
C7—N1	1.373 (4)	C21—N3	1.399 (4)
C8—N2	1.307 (4)	C21—S2	1.649 (3)
C8—N1	1.395 (4)	C22—N4	1.446 (4)
C8—S1	1.658 (3)	C22—C23	1.496 (4)
C9—N2	1.440 (4)	C22—H22A	0.9700
C9—C10	1.492 (4)	C22—H22B	0.9700
C9—H9A	0.9700	C23—O5	1.193 (4)
C9—H9B	0.9700	C23—O6	1.332 (4)
C10—O2	1.182 (4)	C24—O6	1.454 (4)
C10—O3	1.314 (4)	C24—C25	1.483 (4)
C11—O3	1.447 (4)	C24—H24A	0.9700
C11—C12	1.487 (5)	C24—H24B	0.9700
C11—H11A	0.9700	C25—C26	1.512 (5)
C11—H11B	0.9700	C25—H25A	0.9700
C12—C13	1.484 (5)	C25—H25B	0.9700
C12—H12A	0.9700	C26—H26A	0.9600
C12—H12B	0.9700	C26—H26B	0.9600
C13—H13A	0.9600	C26—H26C	0.9600
C13—H13B	0.9600	N1—H1B	0.863 (10)
C13—H13C	0.9600	N2—H2B	0.871 (10)
C14—C15	1.371 (4)	N3—H3B	0.862 (10)
C14—C19	1.375 (4)	N4—H4B	0.867 (10)
			
C2—C1—C6	120.4 (4)	C15—C16—H16A	120.4
C2—C1—H1A	119.8	C17—C16—H16A	120.4
C6—C1—H1A	119.8	C18—C17—C16	120.4 (4)
C3—C2—C1	120.3 (4)	C18—C17—H17A	119.8
C3—C2—H2A	119.9	C16—C17—H17A	119.8
C1—C2—H2A	119.9	C17—C18—C19	120.8 (4)
C4—C3—C2	119.5 (3)	C17—C18—H18A	119.6
C4—C3—H3A	120.2	C19—C18—H18A	119.6
C2—C3—H3A	120.2	C18—C19—C14	118.4 (3)
C3—C4—C5	120.6 (4)	C18—C19—C20	118.0 (3)
C3—C4—H4A	119.7	C14—C19—C20	123.6 (3)
C5—C4—H4A	119.7	O4—C20—N3	121.3 (3)
C4—C5—C6	120.3 (3)	O4—C20—C19	121.3 (3)
C4—C5—H5A	119.9	N3—C20—C19	117.3 (3)
C6—C5—H5A	119.9	N4—C21—N3	115.1 (3)
C5—C6—C1	118.8 (3)	N4—C21—S2	124.8 (2)
C5—C6—C7	124.3 (3)	N3—C21—S2	120.0 (2)
C1—C6—C7	116.8 (3)	N4—C22—C23	108.8 (3)
O1—C7—N1	121.8 (3)	N4—C22—H22A	109.9
O1—C7—C6	121.0 (3)	C23—C22—H22A	109.9
N1—C7—C6	117.2 (3)	N4—C22—H22B	109.9
N2—C8—N1	116.4 (3)	C23—C22—H22B	109.9
N2—C8—S1	124.2 (2)	H22A—C22—H22B	108.3
N1—C8—S1	119.4 (2)	O5—C23—O6	125.0 (3)
N2—C9—C10	109.5 (3)	O5—C23—C22	124.6 (3)
N2—C9—H9A	109.8	O6—C23—C22	110.4 (3)
C10—C9—H9A	109.8	O6—C24—C25	107.7 (3)
N2—C9—H9B	109.8	O6—C24—H24A	110.2
C10—C9—H9B	109.8	C25—C24—H24A	110.2
H9A—C9—H9B	108.2	O6—C24—H24B	110.2
O2—C10—O3	125.1 (3)	C25—C24—H24B	110.2
O2—C10—C9	123.7 (3)	H24A—C24—H24B	108.5
O3—C10—C9	111.1 (3)	C24—C25—C26	111.9 (3)
O3—C11—C12	108.2 (3)	C24—C25—H25A	109.2
O3—C11—H11A	110.1	C26—C25—H25A	109.2
C12—C11—H11A	110.1	C24—C25—H25B	109.2
O3—C11—H11B	110.1	C26—C25—H25B	109.2
C12—C11—H11B	110.1	H25A—C25—H25B	107.9
H11A—C11—H11B	108.4	C25—C26—H26A	109.5
C13—C12—C11	112.4 (4)	C25—C26—H26B	109.5
C13—C12—H12A	109.1	H26A—C26—H26B	109.5
C11—C12—H12A	109.1	C25—C26—H26C	109.5
C13—C12—H12B	109.1	H26A—C26—H26C	109.5
C11—C12—H12B	109.1	H26B—C26—H26C	109.5
H12A—C12—H12B	107.9	C10—O3—C11	117.6 (3)
C12—C13—H13A	109.5	C23—O6—C24	117.0 (3)
C12—C13—H13B	109.5	C7—N1—C8	127.7 (3)
H13A—C13—H13B	109.5	C7—N1—H1B	120 (2)
C12—C13—H13C	109.5	C8—N1—H1B	112 (2)
H13A—C13—H13C	109.5	C8—N2—C9	124.3 (3)
H13B—C13—H13C	109.5	C8—N2—H2B	118 (2)
C15—C14—C19	120.7 (3)	C9—N2—H2B	118 (2)
C15—C14—H14A	119.7	C20—N3—C21	128.6 (3)
C19—C14—H14A	119.7	C20—N3—H3B	117 (2)
C16—C15—C14	120.4 (4)	C21—N3—H3B	114 (2)
C16—C15—H15A	119.8	C21—N4—C22	123.1 (3)
C14—C15—H15A	119.8	C21—N4—H4B	122 (3)
C15—C16—C17	119.2 (3)	C22—N4—H4B	114 (3)

### Hydrogen-bond geometry (Å, °)

**Table:**

*D*—H···*A*	*D*—H	H···*A*	*D*···*A*	*D*—H···*A*
N2—H2B···O1	0.87 (2)	1.92 (2)	2.617 (3)	136 (2)
N2—H2B···O2	0.87 (2)	2.33 (2)	2.663 (4)	103 (2)
N4—H4B···O4	0.87 (3)	1.97 (2)	2.605 (4)	129 (3)
N4—H4B···O5	0.87 (3)	2.23 (3)	2.671 (4)	111 (2)
C5—H5A···S2	0.93	2.84	3.396 (3)	120
C13—H13B···O4^i^	0.96	2.54	3.329 (6)	139
C14—H14A···S1	0.93	2.78	3.397 (3)	125
C24—H24A···O2^ii^	0.97	2.57	3.441 (4)	150
C26—H26B···O1^ii^	0.96	2.57	3.384 (4)	143

Symmetry codes:  (i) *x*, *y*, *z*−1; (ii) *x*, *y*, *z*+1.
